# Fusing together an outline design for sustained fuelling and tritium self-sufficiency

**DOI:** 10.1098/rsta.2023.0410

**Published:** 2024-10-09

**Authors:** Michael Lord, Iryna Bennett, Chris Harrington, Adam Cooper, Dan Lee-Lane, Adam Cureton, Cameron Olde, Megan Thompson, Dinusha Jayasundara, Toby Meatyard

**Affiliations:** ^1^ UKAEA, United Kingdom Atomic Energy Authority, Culham Campus, Abingdon, Oxfordshire OX14 3DB, UK

**Keywords:** STEP, fuel cycle, tritium, fuel self-sufficiency, breeding, breeder blankets

## Abstract

Ensuring tritium fuel self-sufficiency while maintaining continuous and high-specification fuel flow to the tokamak via a low tritium inventory and controllable fuel cycle is a significant challenge to the STEP plant design. Effective and high-quality fuelling and exhaust design is required to sustain and control a stable plasma, whereas fuel sufficiency is required to prevent depletion of available tritium supply. Concerns regarding the lack of tritium availability preventing continuous tritium import are countered by breeding, where highly energetic neutrons from the core fusion reactions interact with lithium atoms suspended in the surrounding breeder blanket to produce tritium. The compact nature of STEP prohibits the integration of inboard breeder blankets posing a significant challenge for the design team looking to ensure more tritium is bred and made available than consumed within the core plasma. This paper outlines how purposeful technology selection and system architecting has converged on the outline of a conceivable and tritium-capable fuel cycle and breeder blanket design. Before introducing the STEP fuel cycle design outline and summarizing the approach undertaken to address the challenges facing plasma fuelling, key aspects of fuel self-sufficiency are discussed. This includes discussing a proposed helium-cooled liquid lithium breeder blanket and possible technology options for tritium extraction from lithium. Lastly, there is a brief process modelling overview, which emphasizes the central contribution of various employed modelling methods. Reflections on the presented fuel cycle design outline conclude that substantial development work is still required to realize a continuous tritium fuel cycle design and overcome the major challenges posed by tritium and lithium handling. Reflections on the presented breeder blanket design proposal conclude that while many substantial risks and blockers remain to achieve fuel self-sufficiency, high breeding ratios are expected to be achievable with a compact spherical tokamak configuration. Nonetheless, it is recognized that further consideration is required to ensure that the selection of liquid lithium as a breeder medium provides the overall simplest route to a self-sufficient and realizable design.

This article is part of the theme issue ‘Delivering Fusion Energy – The Spherical Tokamak for Energy Production (STEP)’*.*

## 1. Fuel self-sufficiency

STEP shall be fuelled by DT, a mixture of the heavier hydrogen isotopes tritium and deuterium. The high nuclear cross-section offered by DT fusion, relative to other nuclides, is required to generate enough fusion power to counterbalance the parasitic power demands of the wider STEP plant. First plasma operations on STEP will be with hydrogen and deuterium fuelled, but ahead of DT operations, there will be a need for an initial import of a start-up tritium inventory. Once charged with the initial import of tritium and operational with DT fuel, the design intent is that STEP will be tritium fuel self-sufficient. STEP will breed its own tritium fuel via the use of a breeder blanket containing enriched liquid lithium. The tritiated liquid lithium will be continuously circulated through an extraction system to recover the tritium and route it to downstream fuel cycle systems for further processing.

The tritium breeding ratio (TBR) is defined as the ratio of tritium bred over the tritium consumed in the fusion reactions within the plasma core. A first-order assessment implies that a 1500–2000 MW fusion power device with a below-unity TBR of 0.9 would require an input of 8–11 kg of tritium fuel per full power year to support continuous operation. Such a dependency on tritium input for continuous fuelling could limit STEP’s power output, and potential delays in fuel deliveries could risk operational schedules, which would be compounded by the lack of global tritium availability. The STEP TBR lower threshold is 1.10. The lower 1.10 TBR threshold is the minimum value that must be realized in practice to be confident that STEP, operating with DT plasma at 20% utilization, will breed sufficient tritium to match the loss of the consumed fuel within the fusion reactors and generate a slight excess to compensate for radiological decay and tritium retention in materials. There is also a desire for STEP to breed excess tritium to act as a potential fusion fuel exporter to start up other fusion devices. The 1.10 TBR threshold limit definition allows for a regular tritium export equivalent to STEP’s inventory every 5–10 years. An early-stage design TBR of target 1.25 has been adopted to ensure confidence in achieving the TBR 1.10 threshold by adding a margin to counterbalance the expected erosion in TBR during the design definition phase. In 2023, a dedicated project was launched to look at options to elevate as far as practically possible, the STEP TBR. The results of this project concluded that a TBR of 1.17 could be practically achieved for the STEP design.

## 2. Introducing the STEP breeder blanket design

The compact nature of STEP means the incorporation of a sizeable inboard breeder blanket is not possible, meaning outboard breeding must be maximized via:

— Using a breeder material with a high atomic density of lithium,
— Using structural and coolant materials with low neutron capture cross sections,
— Elevating, via enrichment, the Li6 content of the breeder,
— Maximizing as far as practically possible, the ratio of breeder material volume to all other materials, and
— If necessary, the incorporation of a neutron multiplier to boost the neutron economy of the system.

A wide variety of different initial designs for the STEP blanket were explored through parametric sweeps of material volumes, enrichment levels and materials selections including assessing different coolants, breeders and structural materials. These studies, undertaken by the UKAEA tool RaBBIT (Rapid Breeder Blanket Integrated Tool) [1], found the breeder material that, in initial nuclear designs, maximized breeding for STEP, was pure liquid lithium, with a peak design point at 35–45% 6Li enrichment. The studies also revealed that for STEP neutronic conditions, TBR was largely independent of the inclusion of a lead multiplier when using liquid lithium. Hence the choice of liquid lithium as the STEP breeder material was found to give the greatest breeding confidence with the potential for lower blanket complexity by removing the need for multiplier integration [2]. The selection of liquid lithium also had some advantages from a supply chain perspective, namely allowing for lower levels of 6Li enrichment and avoiding the use of beryllium. The expectation of extremely high pumping power for self-cooled liquid metal designs, resulting from magnetohydrodynamic (MHD) effects, as well as a desire to reduce the overall lithium and tritium inventories, led to the decision not to pursue a self-cooled lithium design. To mitigate concerns associated with coolant and breeder reactivity in the case of coolant loss, gaseous helium was selected as the blanket coolant.

Vanadium alloy (indicatively, but not necessarily, V−4Cr−4Ti) was selected as the main structural material for the breeder blanket and the integrated outboard first wall. This material selection was necessitated by the need for compatibility with the liquid lithium breeder to prevent excessive corrosion and liquid metal embrittlement [3,4]. The choice of a material inherently compatible with lithium eliminates the need for corrosion coatings, which avoids the issue of coating degradation that could seed a low detectability failure case ultimately propagating to a loss of lithium containment.

**Figure 1.**
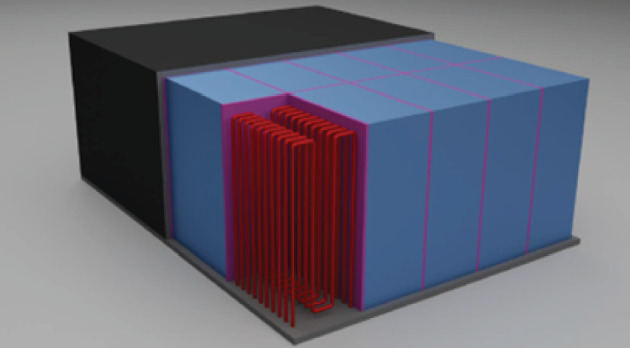
Conceptual illustration of a STEP breeder blanket module including the module box and first wall (larger box at rear of image), lithium breeding zone (shown by 10 tall cuboids with interior of nearest box exposed), helium cooling pipes (M-shaped piping at front of the image) and stiffening plates (sections between the 10 cuboids). The plasma-facing side (first wall) would be at the rear surface in this image.

The general basis for the breeder blanket design proposal is a set of modules featuring a breeding zone containing the slowly flowing liquid lithium intersected by stiffening grids and cooling elements. An illustration of the design proposal is shown in figure 1. The front face of the outer boundary of each blanket module (the upper surface in figure 1) forms the basis of the outboard first wall. In-series cooling is chosen for the first wall and breeding zone: the helium coolant flows first through the outboard first wall to provide cooling, raised in temperature from 400 to ~444°C and is then collected in a manifold and circulated to cool the breeding zone of the blanket, exiting at a temperature of 600°C. This in-series configuration enables greater heat handling capability for the first wall while providing a level of pre-heating to the coolant for the breeding zone, which in turn enables a higher outlet temperature. The coolant is circulated through the breeding zone in U-shaped tubes that are immersed in the liquid lithium. These tubes are small in diameter (~8 mm), which limits direct neutron streaming through the blanket; however, shielding of the outboard vacuum vessel and outboard by the blanket is an important requirement and must be verified through more detailed heterogenous neutronic calculations. The stiffening plates within the blanket modules increase the rigidity of the outer walls. This improves the ability of the blanket to resist internal pressure loads and external loads such as gravity, vibration and electromagnetic loads.

The desired coolant outlet temperature required for net power confidence drives the need for the blanket structural materials to operate at temperatures above around 650°C [5]. Vanadium alloys demonstrate high strength and creep resistance at such temperatures and show a relatively high neutron transparency at 14 MeV neutron energies, which is advantageous to TBR. Nonetheless, data for lithium–vanadium compatibility are limited, particularly in representative environments of 14 MeV neutron irradiation, and there are many failure modes requiring further investigation. This includes improving the definition of temperature limits for low-temperature hardening embrittlement (in this case defined as the region of <400°C) [6] and improving the quantification of creep rates at elevated temperatures (>600°C). There also remains a significant risk associated with sensitivity to embrittlement after permeation or irradiation-induced transmutation of impurities into the vanadium structure [4], which requires expensive testing to fully characterize. Long-term efforts will be required in a testing programme that addresses these key risks, all of which must capture the specific effects of irradiation damage and helium generation under 14 MeV neutron irradiation—this is planned through a combination of fission-spectrum irradiation campaigns, proxy irradiation techniques and accelerated He implantation techniques. Finally, one of the largest blockers to the use of vanadium alloy lies in the undeveloped supply chain. While no showstoppers have been identified, in the absence of other commercial demands for the specific vanadium alloy, this scale-up of the supply chain will require significant investment from the STEP programme.

Further complications stem from the potential for a lithium–water fire or lithium–air fire in the event of loss-of-coolant or loss-of-vacuum accident scenarios. In the former, although water is not used in the blanket, nearby systems such as the divertor are cooled with heavy water. The hazard of a fire with tritiated and activated fumes exists if a common mode failure or a combination of multiple system failures leads to water and lithium interaction. Designing practical, robust and effective preventive systems as well as positive design methods to mitigate the risk of a lithium–water fire is deemed possible but nonetheless represents a major design challenge. It is possible that the safety mitigation systems required to realize a lithium and water tokamak design could result in a prohibitively complex overarching powerplant, counteracting the benefit offered by the comparative simplicity of the liquid lithium design relative to other blanket concepts.

## 3. Tritium extraction from the breeder medium

Fuel self-sufficiency involves not only breeding sufficient tritium but also being able to recover the tritium in a usable form from the medium in which it is bred. This requires:

— A process with minimal tritium residence time, to ensure that the bred tritium can be used to fuel the reactor as soon as possible. This prevents excessive tritium inventories, improves safety and reduces reliance on imported tritium.
— A process with minimal liquid lithium volume. Maintaining the ex-vessel equipment temperature above lithium’s melting point is a power-intensive process and liquid metallic lithium presents a major hazard with little pre-existing industrial precedent on which to base safety measures.
— Minimal reliance on high-temperature operation. The ability of lithium to selectively dissolve most alloying elements already limits the selection of structural materials and designing so to operate at higher temperatures further narrows material options. A design that operates at a high enough temperature to avoid unintentional lithium freezing, but no higher, is preferable.

Tritium extraction technologies for pure lithium are less technologically mature than for other breeder mediums (e.g. solid pebble beds, LiPb or FLiBe) [7]. Technology assessments of the tritium extraction system (TES) remain a work in progress and theoretical assessments of tritium inventory and power consumption have indicated that cold trapping, electrolysis and liquid-liquid extraction (also referred to as the Maroni process [8]) are the most feasible candidate technologies for STEP. Further process modelling and experimental proof-of-concept work must be carried out to improve confidence in the scalability of these processes. The necessity of a dedicated research and development campaign has been identified, including a modelling campaign to explore the viability of candidate technologies. After modelling the use of yttrium getter beds, the technology was rejected owing to the high tritium inventory required in the TES to deliver the required extraction performance.

Another key outcome of STEP tritium extraction work was a targeted assessment of the recommended allowable tritium in lithium concentration in the blanket. Designing for a limit of 5–10 ppm, higher than the 1 ppm concentration common in the literature [9], significantly reduces the required flow of lithium to the TES and hence the size of the extraction equipment. In addition to the reduced likelihood and severity of a lithium leak, smaller volumes of ex-vessel lithium can dramatically reduce the capital expenditure on enriched lithium required to fill the ex-vessel systems, with net savings of the order of £100M.

As part of the wider challenge of lithium handling, the lithium must also pass through a series of purification sub-systems. The purification system will act to remove other elements and compounds present in the lithium, primarily nitrogen, oxygen, carbon and solids such as corrosion products. The presence of nitrogen significantly increases the rate of corrosion and corrosion products themselves may be abrasive and can become activated under repeated neutron bombardment. The lithium purification system will consist of cold traps for the removal of oxygen and carbon and hot traps to reduce the nitrogen levels by contacting the flowing lithium with a titanium mesh. Filters will be employed for the removal of solid corrosion products and will be necessary to prevent lithium pump deterioration. Although technology options for lithium pumping are still under consideration, initial assessments of existing capabilities have shown that the technology is available for commercial-scale flowrates, using either centrifugal or electromagnetic-type pumps [10]. Much insight can be drawn from sodium fast breeder reactors and some pilot plant scale experiments with lithium [11].

The measurement of tritium in liquid lithium is also a significant challenge and is a function that is required for effective process control, safety assurance and tritium accountancy in lithium systems. Batch techniques, though potentially more technologically mature, have many drawbacks owing to the need for sample handling equipment, additional safety hazards, lengthy analysis times and the formation of secondary radioactive waste. *In situ*, continuous monitoring of tritium concentration in lithium is the ideal solution but the maturity of potential technology options is low and the operational environment is challenging. The STEP fuel cycle intends to first demonstrate the capability of an accurate batch-wise technique for the measurement of tritium in lithium, likely via pyrolysis [12] or a wet chemistry approach. Successful development of this technique will enable further technology maturation into continuous tritium in lithium sensors, with dynamic permeation sensors and electrochemical methods identified as preferred options.

## 4. Fuelling

The fuel cycle–plasma interface is a primary fuel cycle interface and requires cross-discipline working to maximize the effectiveness of core fuelling, but also to manage the high heat loads in the divertor exhaust [13]. Moreover, an appreciation of complexity is required to understand and then satisfy plasma control requirements [14], ensuring that fuel and gas injection does not seed or exacerbate plasma instability. It is recognized that fuel and gas injection must support and not hinder plasma control and alongside magnetic profile shaping and heating and current drive, is one of three of the main plasma control actuators.

The DT fuel shall be injected into the plasma core in the form of cryogenic pellets. The solid cryogenic DT pellets, seeded with a small fraction of xenon, will be prepared in an extruder before being propelled by centrifuge along fuelling tubes into the inboard of the STEP tokamak. Centrifuges, backed by screw extruders, were selected over gas guns for core fuelling to satisfy the short-lived but high core fuelling rate, required during the start-up transition from low to high core density plasma.

Fuel injection via the inboard is required to maximize fuel-to-core penetration, which is important for maximizing fuelling efficiency but also for ensuring plasma stability. Greater core penetration means the localized density increase at the edge of the plasma core (where disruptions commonly seed) is reduced. Xenon seeding is required to radiate the heat away from the plasma core to reduce the power in the plasma flowing into the primary divertor. Xenon can be readily extruded in a DT mix [15] and although the most expensive noble gas, owing to its good radiative properties, it can be injected in much smaller amounts than other gases reducing the waste profile of activated seeded impurities.

Fuel pellet penetration has been optimized. A design of experiments parameter space mapping was generated to characterize the impact injection velocity, pellet size, aspect height of pellet injection and angle of pellet injection.

**Figure 2.**
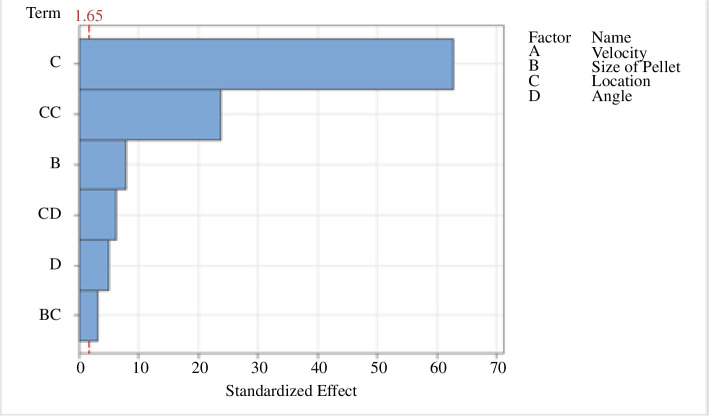
Pareto chart of parameters impacting fuel pellet penetration past the plasma edge (defined as the outer 5% density shell of the plasma core). The chart includes individual and second-order polynomial square interactions with insignificant factors removed.

The JINTRAC model performance outputs were entered into the statistical software package Minitab^®^ to generate a surrogate model such that fuel-to-core penetration could be further optimized with minimal computation effort. The optimization study concluded, refer to figure 2, that penetration is largely governed by the injection location and thus plasma drift effects, reinforcing the need for inboard injection.

Another example of integrated work between the fuel cycle and plasma is the development of a successfully integrated divertor and exhaust plasma scenario. The main system flow of the STEP fuel cycle is not characterized by the core fuel injection but instead by the gas flows injected into the divertor and the plasma edge to control the position of the plasma detachment front and manage divertor heat fluxes via radiative cooling from line radiation associated with electron transition.

An integrated architecture has been developed, which accounts for the complex tripartite relationship between (i) fuelling and gas injection, (ii) vacuum pumping and (iii) the divertor plasma. The generation of a successful integrated divertor scenario requires optimizing the interplay between neutral pressure and impurity concentration. High neutral pressure enhances momentum loss in the divertor plasma but can increase the main chamber pressure, potentially degrading core performance. Conversely, high impurity concentration improves radiation for energy dissipation but risks migrating impurities into the plasma core, diluting the main fuel ions. Therefore, it is crucial to balance these factors to effectively mitigate the heat load on the divertor target plates without compromising overall plasma performance.

To help balance these often-competing requirements, the engineering-constrained design space was characterized using preliminary values of pump capacity scaled as a function of inlet temperature and pressure. Bounded but explorative plasma studies have been undertaken to investigate the impact of key design trade-offs and co-variances for a range of set target divertor pressures with argon-seeded DT cooling gases. These plasma studies focused on characterizing the divertor pressure and then deriving the associated effective pump speed and gas injection rate required to find options to achieve a detached solution. Simple estimations of the detachment threshold show that the divertor pressure should be >10 Pa to keep the argon seeding compatible with core requirements [16]. These divertor pressures are associated with an assumed circumferentially uniform vacuum pumping rate of 20–30 m^3^ s^−1^ and correlate to a total DT injected flow in the region of ~4 times the core fuelling flow. Hence, the pump capacity and divertor pressure requirements set the main fuel cycle flow, not the core fuelling requirement. Gas-puffing DT (opposed to deuterium) into the divertor helps with rebalancing the DT ratio of the unspent fuel. Argon was selected as the seeding impurity for the divertor region because it is chemically inert, gives an acceptable performance as a divertor radiative species and, unlike neon, is readily separated from hydrogenic fuel.

## 5. The wider fuel recirculation system

The fuelling and exhaust systems are both sub-systems within the wider fuel recirculation system. The main fuel recirculation system functions include:

— Fuelling the plasma (covered in the previous section),
— Removing matter from the tokamak vacuum vessel,
— Processing the exhaust streams to remove impurities, and
— Processing and rebalancing the recovered unspent fuel to ensure an acceptable fuel specification and DT ratio prior to re-injection into the tokamak.

The vacuum system’s main function is to reduce the vacuum vessel pressure to ultrahigh vacuum to minimize background impurities that interfere with the core fusion reactions. Owing to the ultrahigh vacuum requirements of the plasma vessel, the inlet pressure into the pump chain will be very low (between 0.0001 and 1 Pa) such that ultrahigh vacuum pumps are required. The vacuum systems will consist of 24 cryogenic pumps, operated in repetitive adsorption and regeneration batch cycles of approximately 30 min. The cryopump design consists of multiple stages of different cryogenic temperature panels. The argon will be captured by a single panel, at the cryopump inlet. This panel will operate during the adsorption phase at 50 K. The DT fuel will be captured via a series of 15K panels coated with activated charcoal. A head of helium and remnant unspent fuel will build up in the cryopump and be pumped out via downstream turbomolecular pumps, backed by mechanical boosters and roughing pumps. These series of pumps will form a vacuum pump chain to extract the unspent fuel and undertake the bulk separation of exhaust species.

It is expected that the bulk exhausted unspent fuel will be captured within the cryopumps. Once regenerated, this stream of unspent fuel will flow into a palladium–silver membrane cascade. In the membrane cascade, the hydrogenic species are separated from non-hydrogenic species. This means the bulk unspent fuel can be quickly recycled. The STEP fuel cycle design outline architecture has the divertor gas supply predominantly fed by the membrane cascade while the core fuelling is fed from (i) recovered tritium bred in the blankets and (ii) the DT streams from the isotope adjustment streams. This architecture has many subtle advantages in terms of fuel cycle controllability.

While the bulk unspent fuel is recycled for divertor gas injection, most of the remaining unspent fuel will flow via the plasma exhaust processing (PEP) system to the isotope adjustment system (IAS). The PEP processes two different streams: (i) primarily hydrogenic fuel and helium that has flowed through the cryopump and (ii) a mixture of activated intrinsic and seeded impurities. The first stream will again be passed by a palladium–silver membrane cascade before flowing into the gas detritiation sub-system for further detritiation. The second stream will consist of a mix of activated argon and xenon. Owing to a desire to minimize radiological discharge, the current design intent is that both argon and xenon will be recovered and recycled, but the capability shall be retained to occasionally purge and discharge fractional volumes. Occasional purges will mitigate the accumulation of large quantities of chemically active, tritiated and radiological species caused by repeat neutronic bombardment of already activated by-products. The seeded impurity exhaust streams shall be radiologically cooled in activated charcoal adsorption beds (where Xe will be cooled for 10s of hours) and decay tanks (where Ar will be cooled for 1000s of hours), which both work via temporary storage of the activated materials while radioactivity levels reduce with time. Architecting of the seeded impurity management systems is a work in progress and candidate technologies include, yet are not limited to, scrubbers, condensers, passivation columns and adsorption filters. Candidate technologies for argon from xenon separation include cryogenic distillation and cryotraps.

Protium can build up in the fuel cycle from material off-gassing and drift in the DT fuel ratio can develop over time owing to slight differences in the time lag between the different hydrogenic processes. The IAS aims to remove and purge protium from the unspent fuel and to provide a high-purity tritium stream, which can then be used to stabilize the fuel DT ratio. The IAS will primarily consist of many parallel thermal cycling absorption processing (TCAP) units [17]. These units will be established in a modular architecture to minimize individual unit tritium inventory, but also to support ease of system upgrade or maintenance. The modulated design approach will also offer flexibility for modification during operations as functional requirements may change with plant life, for example, the volume of hydrogen outgassing will vary with time as new components are introduced to the operational system. Moreover, pressure swing adsorption processes or quantum sieving could be employed, as upgrades or options, in a modulated system that enhances sub-system performance or primes the TCAP inlet.

TCAP units separate hydrogen isotopes based on different isotopic affinities to absorbents and adsorbents in a chromatographic semi-continuous process. The system is configured with two columns separated by a single valve. One column is filled with palladium on a substrate that preferentially absorbs lighter isotopes (H > D > T). Another column, named the inverse column, is filled with molecular sieve adsorbents that preferentially absorb heavier isotopes (T > D > H). The column temperatures are swung to result in the enrichment of tritium in the product stream.

One main challenge to the development of the IAS is the industrialization of TCAP units, both in terms of increasing unit size and building supply chain capability to deliver many units. The use of TCAP has many advantages over the main technology alternative, cryogenic distillation, but is only possible owing to a low inlet flow. The successful incorporation of the TCAP units, specifically maintaining or improving on the number of TCAP units in the system, depends on minimizing the fraction of the plasma exhaust sent to the IAS. This is determined by minimizing protium ingress and optimizing the performance of the vacuum and membrane sub-systems.

## 6. The tritium recovery system

The TRS’s main function is to provide detritiation to a series of working fluids and gas streams to maximize tritium containment within the fuel cycle and minimize radiological release to the environment.

The trace tritium recovery (TTR) recovers tritium from remanent in the outflows from the IAS. The tritium is recovered as a diatomic gas. The TTR will contain a few large cryogenic distillation columns, supported by equilibrator reactors, downstream of the IAS TCAP units. Equilibrators are strategically placed to enable the conversion of HT into a form that enables recovery of the tritium while also removing the protium. The decision to have a series of TCAP units, in the IAS, backed by a cryogenic distillation column in the TTR, as opposed to a series of cryogenic distillation columns spanning both systems, was to minimize the inventory of hydrogen in a liquid state and to avoid control issues associated with a series of columns.

The gas detritiation system ensures tritium release to the environment is minimized via the detritiation of the multiple gas exhaust systems *en route* to the stack. The selected method to maximize recovery is the conversion of tritiated species to water, by catalytic recombiners, from which the tritiated water is then removed from the gas stream via a molecular sieve bed. The tritiated water is then fed to the water detritiation system for further processing and the remaining detritiated gas is routed to stack for release into the environment.

The water detritiation system (WDS) recovers tritium from water generated within the tritium recovery loop of the fuel cycle. The WDS outputs detritiated water to be discharged to stack or potentially used in other parts of the fuel cycle or wider STEP plant (aka as a coolant). The WDS is also capable of removing protium from the fuel cycle. The selected technology for the WDS is a combined electrolysis and (liquid phase) catalytic exchange (CECE) [18]. The liquid phase catalytic exchange column contains packing to maximize contact between the down-flowing water and the up-flowing hydrogen gas (produced by the electrolyzer).

In addition to the detritiation of the exhaust streams *en route* to the stack, the TRS shall contain multiple sub-systems for the detritiation of the plant and fuel cycle coolants and working fluids. The main coolants requiring detritiation are the heavy water and helium coolants. Tritium primarily ends up in the coolant systems via permeation, and transmutation, but also owing to breeding in the heavy water. Zirconium alloy getter beds will be employed to detritiate the helium coolant [19]. Multiple beds can be used in parallel to ensure continuous operation during regeneration cycles. An upstream non-regenerable getter is implemented to capture gaseous impurities such as oxygen and nitrogen, to avoid poisoning of the getter beds. For the detritiation of the heavy water coolant, water distillation followed by CECE will be employed. The water distillation column produces an offtake stream of tritiated water that will be sent to a CECE system for heavy water detritiation, while the detritiated water is passed back to the wider coolant system. Distillation is a proven technology across multiple industries and there exists a long-standing capability of handling tritiated water in large quantities as part of nuclear fission operations in Canada [20], nonetheless, challenges and risks remain as well as concerns regarding the power required to continuously heat large volumes of water.

With heavy water as one of the main STEP coolants, there is an intrinsic issue of needing to handle and manage large volumes of low-grade tritiated water. Although this introduces a distinct hazard, the detritiation and handling of tritiated water is deemed much easier to resolve, via the application of water distillation columns, than the detritiation and handling of other coolant alternatives. Moreover, the inclusion of water detritiation capabilities into the fuel cycle complements and aligns with the application of high detritiation factor technologies in the gas detritiation blocks/sub-systems.

The air detritiation system (ADS) handles very low tritium levels in air caused by the small quantities of tritium that have permeated out of the fuel cycle secondary containment into the fuel cycle facilities. The ADS has been broken down further into two sub-systems: the purge detritiation system (PDS) and the vent detritiation system (VDS). The PDS focuses on the detritiation of containment spaces, such as gloveboxes and coaxial pipework; the VDS detritiates the room air passing through the various facility heating ventilation and air-conditioning (HVAC) systems. Catalytic recombination and water scrubbing have been downselected as the primary technologies for the detritiation of HVAC systems. For the purposes of HVAC detritiation, wet scrubber columns have been selected over molecular sieve beds owing to a greater ability to process continuous large flowrates and robustness to impurities. Recombiners and passivation columns shall also be employed to remove hydrocarbons, tritiated water vapour and halogens, which may be prevalent in fire situations.

A single system is not expected to process all streams together, owing to large flow rates, differences in contamination levels and/or locational constraints. The plant rooms will be partitioned and categorized into zones based on the expected tritium contamination level and HVAC detritiation requirement. More broadly, the current uncertainty regarding the definition of off-normal events, which will likely size the ADSs, may lead to oversized ADSs for the STEP prototype powerplants. One further technical risk associated with oversizing the system, in addition to large system cost and plant integration, is ensuring that the ADSs provide high performance when presented with normal operational inlet conditions and when present with, potentially very different, off-normal transient or fire case conditions.

## 7. Examples of fuel cycle modelling applications

Modelling is essential to the development of process plants, but modelling tritium fuel cycle design is especially challenging. Modelling provides a means to translate rig and experimental measurements into a full-scale integrated design. It also enables structured sensitivity analyses and optioneering studies. Modelling capability supports sub-system design activities and provides a basis for a robust integrated design philosophy. It provides an improved understanding of how sub-systems operate, how they impact adjacent systems and how they respond to their inlet conditions and environment. Hence, developing modelling capability is essential for addressing many of the key challenges of fuel cycle design. However, modelling a fusion fuel cycle is not a customary task and poses a unique set of challenges, including modelling a suite of low-maturity technologies without an established set of physical property correlations.

### (a) Property environment tool: providence

**Figure 3.**
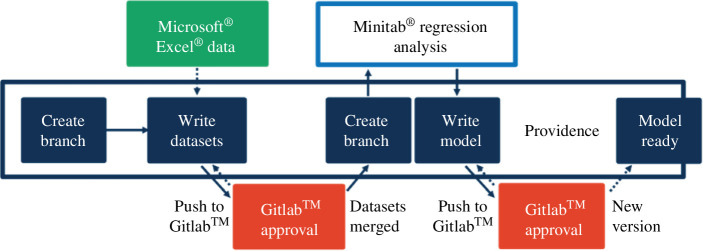
Outline of the Providence database structure.

A principal challenge is sourcing or generating a reliable physical property environment, comprising a coherent system of thermodynamic models that describe the fundamental behaviour and interactions of the constituents within the various process streams. A literature search found a large variation in available published physical data. While Souers [21] has documented extensive and high-quality datasets for the physical properties of hydrogen species for use in fusion applications, data from other sources were largely found to be inconsistent and incomplete. To address this shortfall, all the available raw data were collated and subject to regression analysis to simulate parameters but also generate confidence intervals associated with each fitted constant. A best-fitting regression form was chosen for each property dataset using forms readily available in Microsoft Excel. The raw data, correlations and confidence intervals were built into a standalone database named Providence, refer to figure 3, which is stored in json format and is therefore accessible from a wide range of applications.

### (b) Modelling hydrogen transport

The transport of hydrogenic species through solid barriers has been well researched but remains a phenomenon with many poorly characterized aspects including large experimental uncertainty. A modelling strategy to develop predictive capability across a wide range of fuel cycle applications that encapsulates micro- and macroscale phenomena remains a work in progress. Hydrogen transport modelling must include accurately replicating the behaviour of hydrogens at the solid surface and it has been determined that this behaviour could not be adequately represented by a single Sievert’s constant. A multiparameter approach was therefore developed using a sweep of surface kinetics as boundary conditions, and the method was matched against permeation rates through 316 stainless steel and across palladium–silver membranes. Further work is focusing on introducing trapping within the metal bulk. It is intended to eventually link observable bulk features, such as defects owing to radiation damage to trapping parameters within the model. The ability to quantify the evolution of retention of tritium within the first wall is key to confirming the tritium inventory of the plant and the TBR required to attain fuel self-sufficiency. Quantifying the permeation rates and retention levels of tritium in fuel cycle components and pipework will also be central to evaluating the expected fuel cycle waste profile.

### (c) Cryopump modelling: an example of dynamic modelling

Many fusion fuel cycle technologies have a low level of technical maturity with minimal operational experience to support both steady-state and dynamic model development. Furthermore, dynamic modelling strategies typically require development from first principles. A complex and important modelling challenge is the dynamic modelling of the vacuum cryopump. Cryopump performance has a significant impact on divertor heat management, plasma control as well as the operation, sizing and dynamics of the fuel cycle. Other fusion and industrial applications where cryopumps have been previously used are all within batch processes where dynamics are not critical. A new modelling approach is being developed incorporating low-pressure flow simulations and a means to integrate the cryopump dynamics with the rest of the fuel cycle. The newly developed STEP models have been fitted to a wide range of experimental data from the JET active gas handling system and will be capable of predicting performance in both regeneration and adsorption modes.

### (d) Proving modelling capability: validation

The level of confidence in the models is in large part based on the quality of the underlying validation data and data analysis. The sources of the data used for the development and validation of the models include relevant literature sources, measurements from past experiments and, where appropriate, inference from available operational data. The UKAEA is in a unique position of ownership of and access to facilities not widely available elsewhere in the industry. These include notably the JET active gas handling system and the MAST-U experiment. Several new unique facilities are currently either in design or in construction. In addition to these UKAEA owns and operates several rigs, which focus on important aspects of fuel cycle operation. Some of the experiments are dedicated specifically to obtaining parameters for the models, however, most have other goals, not related to fuel cycle model development. Statistical analysis methodologies are implemented to ensure the best use of the resources and extract the best value from the valuable experimental data. Tools and methods such as design of experiments, uncertainty quantification and regression analysis constitute a key part of modelling capability.

## 8. Fuel cycle tritium inventory and first wall retention

First, it is noted that the safety considerations associated with the fuel cycle design outline and tritium handling are covered as a case example in the STEP discussion on safety [22]. Part of improving plant safety is minimizing the plant tritium inventory. Although the STEP fuel cycle design has looked where possible to minimize tritium inventory, it is not expected that a <1 kg tritium inventory (as desired by some fusion industry fuel cycle architects) is deemed viable for the existing STEP plant architecture. The STEP fuel cycle tritium inventory required to start up the plant is expected to be of the order of several kilograms. Design development of the containment systems for the releasable inventory (fraction of the total inventory) is a work in progress. Further work is also required into the time evolution nature of tritium inventory, particularly surrounding tritium retention in the first wall. Tritium inventory retained within the first wall has the potential to be one of the primary contributors to the plant inventory and thus accurate modelling of first wall retention is key to establishing the expected STEP tritium inventory. In addition to developing a strategy for modelling tritium retention in neutron-damaged walls, the STEP fuel cycle team is developing a scenario mapping tool, based on a standard robust engineering design methodology, to map both useable and retained tritium volumes. Specifically, a design of experiments framework has been generated that can effectively translate how parameters, including but not limited to tritium first wall retention, divertor gas flows, plant availability and wall conditioning profiles, may impact the minimum required TBR and estimate tritium inventory ranges through the initial years of STEP operation.

When the STEP plant is operational, there will be a requirement to accurately monitor first wall retention, which will be critical to ensure adequate and precise tracking of tritium. This is technologically challenging owing to the inaccessibility of first wall components and the harsh environment of the tokamak. The STEP fuel cycle therefore intends to deploy a suite of *ex situ* and *in situ* measurement techniques to characterize first wall retention. The use of laser-induced desorption mass spectrometry (LID-MS), a technique that has recently been successfully deployed in JET and laser-induced breakdown spectroscopy, will provide a complementary means of surveying first wall components for tritium content.

## 9. Conclusion

**Figure 4.**
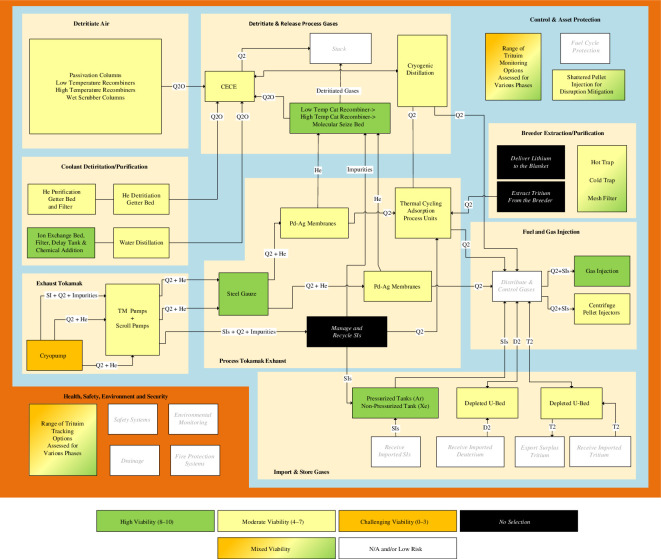
Illustration showing the technology selections for the STEP fuel cycle via block and grouped within functional families. The technology selections have been scored against a viability score devised for the purposes of establishing a feasibility scoring of the technology being able to develop and mature in time for STEP programme requirements.

Technologies have been selected for most STEP fuel cycle functional blocks, figure 4. The high-risk functional blocks are lithium management (including tritium in lithium measurements), tritium extraction from the breeder, vacuum pumping and the processing of seeded impurities. Furthermore, there remain multiple challenges associated with the use of helium-cooled liquid lithium blankets, including but not limited to the scalability of the vanadium supply chain, the safety aspects associated with adjacent lithium and water systems and the integration of the helium cooling lines. Nonetheless, no showstoppers have yet to be identified for fuel self-sufficiency or the use of liquid lithium as a breeder medium. While the wider STEP plant design converges onto a unified design concept, the STEP design team will continue to consider whether liquid lithium remains the best available option or whether there are design paths available that trade an acceptable level of reduction in expected TBR for a greater reduction in design and programme risk. Work will continue to better characterize the correlation between tritium first wall retention, the frequency of wall conditioning processes and the minimum required TBR to ensure the long-term availability of tritium fuel. To summarize, tackling the challenge of fuel self-sufficiency and breeding will be undertaken in parallel with wider fuel cycle design definition, technology derisking and development of the modelling tools required to deliver a fusion fuel cycle. The UKAEA is the home of the tritium fuel cycle division, which is dedicated to addressing the scientific and technological challenges associated with the use of tritium for fusion fuelling. The STEP fuel cycle design will continue to benefit from partnership with the UKAEA and strategic engagement with the wider fusion industry.

## Data Availability

Additional data are available as supplementary material [23].
